# Feasibility of an Outpatient Training Program after COVID-19

**DOI:** 10.3390/ijerph18083978

**Published:** 2021-04-09

**Authors:** Martina Betschart, Spencer Rezek, Ines Unger, Swantje Beyer, David Gisi, Harriet Shannon, Cornel Sieber

**Affiliations:** 1 Institute of Therapies and Rehabilitation, Kantonsspital Winterthur, 8400 Winterthur, Switzerland; spencer.rezek@ksw.ch (S.R.); ines.unger@ksw.ch (I.U.); david.gisi@ksw.ch (D.G.); 2 Department of Medicine, Kantonsspital Winterthur, 8400 Winterthur, Switzerland; swantje.beyer@ksw.ch (S.B.); cornel.sieber@ksw.ch (C.S.); 3 Department of Physiotherapy, University College London, London WC1N 1EH, UK; h.shannon@ucl.ac.uk

**Keywords:** COVID-19/SARS-CoV-2, outpatient pulmonary rehabilitation, physiotherapy, feasibility

## Abstract

Long-term physical consequences of coronavirus disease 2019 (COVID-19) are currently being reported. As a result, the focus is turning towards interventions that support recovery after hospitalization. To date, the feasibility of an outpatient program for people recovering from COVID-19 has not been investigated. This study presents data for a physiotherapy-led, comprehensive outpatient pulmonary rehabilitation (PR) program. Patients were recruited after hospital discharge. Training consisted of twice weekly, interval-based aerobic cycle endurance (ACE) training, followed by resistance training (RT); 60–90 min per session at intensities of 50% peak work rate; education and physical activity coaching were also provided. Feasibility outcomes included: recruitment and dropout rates, number of training sessions undertaken, and tolerability for dose and training mode. Of the 65 patients discharged home during the study period, 12 were successfully enrolled onto the program. Three dropouts (25%) were reported after 11–19 sessions. Tolerability of interval-based training was 83% and 100% for exercise duration of ACE and RT, respectively; 92% for training intensity, 83% progressive increase of intensity, and 83% mode in ACE. We tentatively suggest from these preliminary findings that the PR protocol used may be both feasible, and confer benefits to a small subgroup of patients recovering from COVID-19.

## 1. Introduction

It has been predicted that approximately 45% of patients discharged from hospital after suffering coronavirus disease 2019 (COVID-19) will require further support from the healthcare system [1]. To date, longer-term consequences were found in respiratory and cardiopulmonary function [2], fatigue [3] and quality of life [4,5]. Limitations in physical performance could also be inferred, according to a recent review on long-term consequences observed in patients after severe acute respiratory syndrome (SARS) and Middle East respiratory syndrome (MERS) [6]. However, evidence related to changes in physical performance specifically after COVID-19 remains limited. Two observational studies reported that approximately a third of patients had low physical performance at discharge [7] and after three months [8] following hospitalization for COVID-19. Physical performance was quantified using the 6-min walking test (6MWT). Distance covered during the 6MWT was found to be significantly reduced after four months in survivors of COVID-19 with severe and critical disease, compared to those with a mild or moderate course (*p* = 0.001; not-adjusted for age and gender) [9].

World physiotherapy guidelines for physiotherapy input from the acute stages of COVID-19 to post-discharge were issued in an attempt to reduce the likelihood of long-term physical impairments in the COVID-19 patient population [10]. The post-discharge guidelines included specific information on dose (i.e., frequency per week and repetitions) for aerobic exercise, resistance, balance and training in activities of daily living. Recommendations focused particularly on progressive home-based exercises. The outcome of such training is still unknown although, even in the early phase of the pandemic, expert opinion suggested the need for specific rehabilitation and systematic follow up in patients after hospitalization [11,12,13,14]. More specifically, these papers suggest a form of comprehensive outpatient pulmonary rehabilitation (PR) for the anticipated respiratory impairments, muscle atrophy associated with physical deconditioning as well as other negative consequences of COVID-19, including fatigue and hospital-induced anxiety or depression. PR is characterized by its comprehensive approach that includes exercise training, education and behavior change [15]. To date, there is still a lack of data about the feasibility and outcome of post-discharge rehabilitation as summarized by the rapid evidence paper [16]. Questions posed in April 2020 remain, including whether specific intensities are tolerated by patients post-COVID-19 [12] as recommended in PR programs [17]. Recently published reports showed that interval training was well tolerated in patients after critical and severe COVID-19 attending a comprehensive inpatient rehabilitation [18,19]. These authors suggest the importance of a comprehensive program based on their findings.

To our knowledge, no reports have been published on the feasibility of outpatient PR programs after COVID-19 including moderate-intensity exercise. This paper aims to present feasibility data of outpatient pulmonary rehabilitation. This includes recruitment rate, adherence and tolerability to the training intervention. Additionally, the paper will provide preliminary data on the outcomes of outpatient PR with specific information on dose and training intensities.

## 2. Materials and Methods

The study explored the feasibility of an existing PR program, focusing on the new (COVID-19) population. Patients who had been hospitalized with COVID-19 in one acute hospital setting in Switzerland, were eligible for the program. Patients who had been discharged home between March 2020 and June 2020 were contacted by phone as part of a physiotherapy-guided aftercare program. Patients were invited to undertake an evaluation of physical performance and quality of life conducted by physiotherapists specializing in PR. According to the assessment findings and pre-defined inclusion criteria, patients were offered participation in the outpatient PR program. Data from the assessments and training were recorded in the hospital’s software as per usual care. These data were collected and treated according to the scope of an observational study approved by the local ethical committee (2020-00899). The study was registered at ClinicalTrials.gov (NCT04375709). All patients signed a general or informed consent form approved by the local ethics committee, and could withdraw from the study at any time.

The following aspects of feasibility were evaluated, based on the work of Thabane and colleagues (2010) [20] and Nilson et al. (2018) [21]:

*Recruitment rate.* The percentage (%) of patients meeting the inclusion criteria for outpatient PR from the entire cohort, and % of patients participating from the potentially eligible cohort. Dropout rate (%) with dropouts defined as patients ceasing training due to (1) an adverse event or (2) patient preference to discontinue training before individual goals in physical performance and quality of life were reached.

*Adherence.* The number of training sessions attended, as a percentage of the recommended number of sessions to achieve individually tailored goals for physical performance and health-related quality of life (HRQoL). The expected duration was 16–24 sessions, according to the recommendations for PR in patients with chronic obstructive pulmonary disease [22].

*Tolerability.* Percentage of patients requiring a reduction in training frequency (sessions per week), training intensity (during the session or before session begin), training duration (interval-based cycle endurance (ACE) and resistance training (RT)), or a change in exercise mode of the ACE (from interval to continuous). Number of adverse events due to the training as defined by the Office of Human research Protections (OHRP; USA) [23]. Expected adverse events were not predefined.

Tolerance of training mode and intensity was expected in all patients based on the findings of inpatient PR programs after COVID-19 [18,19].

Physical performance was tested with the 6-min walk test (6MWT) according to the American Thoracic Society (ATS) guidelines [24]. Degree of disability posed by breathlessness was calculated using the 4-point ordinal modified Medical Research Council (mMRC) Dyspnea Scale [25]. HRQoL was assessed using the Euroqol 5-level EQ-5D (EQ-5D-5L) visual analogue scale (VAS) The EQ-5D-5L VAS ranged from 0–100% with 100% representing “the best health you can imagine”. Perception of COVID-19 specific limitations in daily life was quantified using the Post-COVID-19 Functional Status scale (PCFS) [26].

Post-viral fatigue was quantified with the fatigue severity scale (FSS) [27]. The presence of hospital-related anxiety, depression and the risk for post-traumatic stress were screened using the Hospital Anxiety and Depression Scale (HADS-D/-A) [28], and Impact of Event Scale-Revised (IES-R) [29], respectively. Severity of pneumonia was categorized according to the interim guidance of the World Health Organization (WHO) into mild (1), moderate (2), severe (3) and critical (4). All assessments were conducted on-site, 2–9 days before the training program and post-training. Patients were offered outpatient PR if they met the following inclusion criteria: (a) confirmed diagnosis of COVID-19 with at least one positive SARS-CoV-2 nasopharyngeal swab (b) discharged home from study site after hospitalization (c) at least 14 days after confirmed diagnosis of COVID-19 and at least 4 days without COVID-related symptoms (fever [axillary temperature > 37.3 °C] [21], sore throat, cough (productive or non-productive related to COVID-19) or common cold; (d) presenting at least one of the following clinical indicators during baseline evaluation post-discharge. Moreover, clinical indicators were, (1) distance covered during the 6MWT (6MWD) two standard deviations below the age- and gender-specific norms (<80%) or a 6MWD below the lower limit of normal [22]; and (2) perceived COVID-19 related limitations of functional independence in daily life (Post-COVID Functionality Score [PCFS >1]) [19], EQ-5D-5L VAS < 80% [23].

Patients with (1) severe post-viral fatigue (≥4) [20], (2) signs of hospital induced anxiety, depression (HADS-A/D > 10) [28] or post-traumatic stress symptoms (IES-R > 0) [29], (3) signs of nutritional deficits (<24), (4) current smoking habits or (5) signs of uncontrolled heart disease (hearth failure according to the New York Health Administration (NYHA) III–IV, arrhythmias, angina pectoris) and lung disease (dyspnea mMRC 3–4, hypoxemia at rest) were discussed with the in-house pulmonologist for decision-making regarding onward referral to other specialists for further evaluation and treatment.

### 2.1. Management during the Pulmonary Rehabilitation (PR) Program

The PR included initial goal setting in terms of physical performance and quality of life, exercise training (a combination of aerobic exercise and resistance training), education and physical activity coaching. Based on the recommendations by the American Thoracic Society and European Respiratory Society (ATS/ERS) Task Force on Pulmonary Rehabilitation, individual goal setting was used (1) for enhancing self-efficacy and self-management abilities, and improving outcomes (2) [30,31]. Prior to the start of PR, goals were assessed according to the International Classification of Functioning, Disability and Health (ICF) of the WHO at activity and participation level. Goals were set on basis of the “Specific Measurable Accepted Realistic Time Bound” (SMART) criteria [32].

To provide individualized, comprehensive training, responses to exercise and physical or psychological limitations were discussed with an in-house pulmonologist once per week. Onward referral or additional assessments (such as cardiac, lung function, sleep studies or musculoskeletal assessment), were undertaken as deemed necessary by the physiotherapist or physician.

#### 2.1.1. Determination of Peak Work Rate

The patient’s exercise capacity was defined with the peak work rate (peakWR; watts), using the incremental, ultra-short maximal steep ramp test (SRT). The SRT is an accurate and reliable measure of exercise capacity in patients with chronic obstructive pulmonary disease (COPD) [33], patients after chemotherapy [34], children with cystic fibrosis [26] and patients with chronic heart failure [35]. Moderate to strong associations have been found between peak work rate during the SRT and the peak oxygen uptake obtained from cardiopulmonary exercise testing (CPET) [34,35,36]. The CPET is considered as the ‘gold standard’ for testing exercise capacity in different populations since it provides a global assessment of exercise response [37]. However, CPET protocols require specific resources to obtain ventilator parameters (i.e., gas exchange and peak or maximal oxygen uptake). The SRT does not test ventilatory parameters and is a practical alternative for everyday clinical use. The SRT is already in use for assessing exercise capacity for patients enrolled in the PR program.

The SRT starts with a three-minute warm-up at 20 watts. Afterwards, resistance is increased by 25 watts every 10 s. The SRT was conducted by physiotherapists specializing in PR, who were aware of possible data use for the purpose of the study. Testing was conducted on computer-guided cycle-ergometers (FREI SWISS^®^, Thalwil, Switzerland), which were also used for the aerobic training described below. Patients were instructed to maintain their pedaling cadence between 60–80 repetitions per minute (rpm). The test was stopped when cadence fell below 60 rpm. During the SRT, heart rate was monitored with heart rate monitoring breast belts connected with the training software (ers.ergoline^®^, Germany).

#### 2.1.2. Training Intervention

Training content, dose and intensity were based on the recommendations for PR, according to Gloeckl and colleagues (2013) [17]. The WRpeak achieved in the SRT is higher compared to the WRpeak by CPET [35,36], therefore intensity levels were adjusted accordingly (Table 1). Patients trained twice a week, guided by a physiotherapist specializing in cardiorespiratory rehabilitation. The minimum number of sessions expected for completion was 16, based on the recommendations of Garvey et al. [22]. Training consisted of a combination of 30 min of aerobic cycle endurance (ACE) training followed by 30–40 min of resistance training (RT). For both modes, intensity was adjusted progressively, reaching a perceived exertion of between 4–6 on the modified Borg Scale (0–10) [38].

The ACE training was a mixed program with 2 sessions of continuous mode followed by two sessions of interval mode. For the continuous mode, initial training intensity was 20–30% of peakWR for 30 min. In the sessions with interval mode, patients cycled at 15% of the maximal work rate (Wmax) during four-minutes for warm-up. This was followed by four bouts of high-intensity and three intervals of moderate-intensity for four- and three-minutes per bout, respectively. The ACE ended with a three-minute cool-down at 15% Wmax. The initial intensity was 50% peakWR for high intensity and 20–30% peakWR of the SRT for moderate intensity, respectively.

During the ACE, heart rate was monitored as described for the SRT. Perceived exertion was quantified using the modified Borg scale. Following a perceived exertion of 5/10 on the modified Borg scale at the end of the ACE exercise at high- and moderate-intensity, resistance was increased by 5 watts for the next training session. For patient safety, blood level oxygen was monitored during the training using finger pulse oximetry. Immediately after the ACE, patients started with the RT. The RT included six device-based (Technogym^®^, Italy) sets to train large muscle groups: chest press, low row, back extension, leg abductor, leg curl and leg press. Each set included 10–12 repetitions at a moderate speed at 50–85% of their one repetition maximum according to the ATS/ERS statement on PR [39] cited in Gloeckl et al. (2013) [17]. Patients repeated the set three times per device. Weight was adjusted when patients achieved more or fewer than 10–12 repetitions.

For safety purposes, as recommended by the infection prevention and control department, cycles were surrounded by plexiglass, which allowed exercise without a facial mask. Further, groups were a maximum of five patients allowing a distance of at least 2 m between the cycles. For RT, patients disinfected each device after usage. During RT patients wore facial masks. Physiotherapists and assessors wore facial masks during the entire training session. During the ACE one therapist observed patients’ performance and vital parameters from a monitor placed at least 2 m distance of the cycles. During RT, patients were verbally instructed.

### 2.2. Statistics

Descriptive statistics are reported as mean (standard deviation [SD]) and 95% confidence intervals (CI), median or frequencies for patients’ demographics, clinical characteristics and feasibility outcomes. For 6MWD age and gender-related norm values were calculated as a percentage of the norm and distance to the lower limit of normal (LLN) according to Enright et al. [40]. The normality of clinical data (6MWD, PCFS, EQ-5D-5L VAS) was tested with the Shapiro–Wilk test. The non-parametric Wilcoxon rank sum was applied to analyze continuous variables and Fisher’s exact test for categorical variables for pre-post-training comparison. The level of significance was set at a two-sided *p* < 0.05 for all comparisons. Data were analyzed using IMB SPSS (version 25).

## 3. Results

### 3.1. Participants

Between April 2020 and June 2020, a total of 65 patients were discharged home from the study site after COVID-19 necessitated hospitalization. Among these, 60 patients accepted being assessed in the physiotherapy-led aftercare program. A 6MWT was obtained from 48 patients. Patients were not able to conduct the 6MWT due to orthopedic limitations (n = 4), four patients did not wish to return to the hospital for assessments and provided the HRQoL, PCFS and fatigue questionnaire completed at home, and four did not provide consent for data use. The 48 patients assessed for physical performance (6MWT) presented with mild (n = 9), moderate (n = 23), severe (n = 11) and critical (n = 5) manifestations of pneumonia according to the interim guidance of the World Health Organization [41]. In total, 12 patients participated in the PR program. Patients’ characteristics are illustrated in Table 2.

Duration between COVID-19 diagnosis and the start of PR ranged from 21–73 days. Two patients started 69 and 73 days after the diagnosis of COVID-19. These patients were re-hospitalized after discharge due to internal and musculoskeletal issues. Therefore, baseline assessment was postponed. The HRQoL was below 80% in 10 patients. Nine patients perceived moderate to severe COVID-induced restrictions (PCFS 2 and 3). Eleven patients initially reported dyspnea during level walking (mMRC score 1–2); One patient had no signs of dyspnea.

### 3.2. Feasibility Outcomes

#### 3.2.1. Recruitment Rate

**Figure 1:**
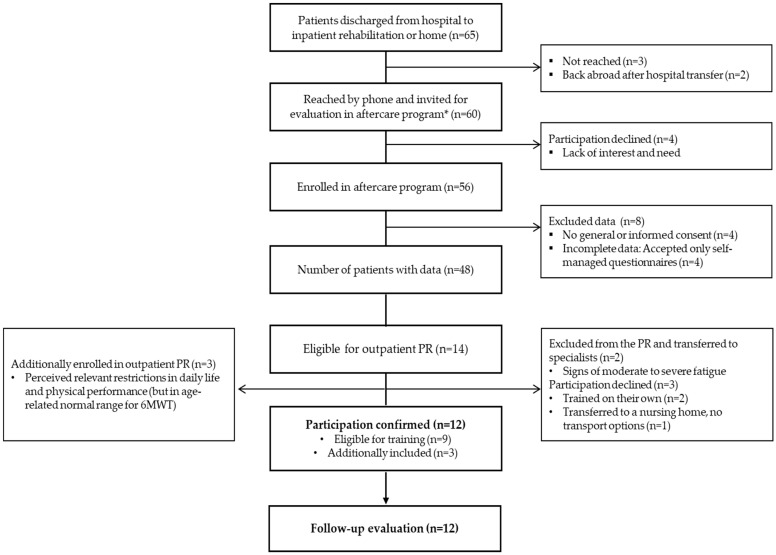
Flow-chart of patient recruitment. PR = pulmonary rehabilitation; * aftercare program = a standard follow-up program developed independently of the research and included testing and counseling of patients post-COVID-19 primarily by specialized physiotherapists and pulmonologists.

Of the 48 patients assessed, 29% (n = 14) met eligibility criteria for the current outpatient PR program (Figure 1). Of these, 64% participated in the training program (n = 9/14). Two patients showed limitations in walking distance due to severe fatigue. They were excluded from PR and were transferred to specialists. Three patients refused to participate. Two of these trained on their own (in a fitness center or at home), and one lived in a nursing home.

Three patients presented normal performance values with the 6MWD (>80% of norm) but perceived relevant restrictions in physical performance and requested participation, which was granted.

#### 3.2.2. Adherence and Tolerability to PR

Eight patients completed 16–25 sessions. Two patients stopped at 12 and 14 sessions respectively, which is earlier than the recommended 16 sessions. These patients stopped because they perceived their performance level and quality of life to have reached the level it was before the infection. These two patients were not considered dropouts.

Three dropouts (25%) were reported after 8, 11 and 19 sessions. These were due to a musculoskeletal issue, planned knee surgery being brought forward and an payment omitted by an insurance provider. All patients completed their post-training assessments.

All 12 participants tolerated two sessions per week for the duration of the program. Training duration for ACE was achieved by 83% of the patients. Ten achieved the requested training duration of 30 min after a range of 2 to 3 training sessions. Two individuals achieved an average of 17 min and remained below the requested 30 min. The reduction in exercise duration enabled these two patients to train in the requested intensity. Exercise duration during AT was tolerated in all 12 patients for the entirety of the program.

Ten patients tolerated the training modes of ACE according to the recommendation. Two patients trained in the continuous mode (17%). One because of chronic activity-dependent leg pain after large-vessel thrombosis leading to the inability to tolerate the intensity of the interval. The other participant trained on an arm cycle ergometer because of pre-existing arthritic knee pain.

Eleven out of 12 patients (92%) were able to start with the recommended intensity of 50% peakWR. Three remaining participants started at 40% of peakWR. A progressive increase in the intensity of training was achieved in 9/12 patients without any negative consequence on physical condition after training. Three patients were not able to progress with intensity because of musculoskeletal pain (n = 2) and weakness induced by a historical renal transplantation (n = 1). All participants tolerated the recommended intensity in RT until the end of the program. One adverse event was observed with a participant experiencing a recurrence of a pre-existing low back pain during the training.

### 3.3. Clinical Outcomes

**Figure 2:**
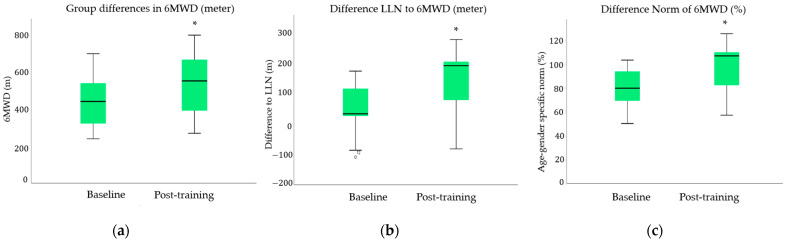
Illustration of clinical data with changes in physical performance: Group mean changes in (**a**) 6MWD (meter), (**b**) difference to the gender-specific lower limit of normal (LLN), (**c**) change in age-gender specific norm expressed in percentages. Statistically significant changes are indicated with the * with *p* ≤ 0.001; Difference Norm of 6MWD (%) was obtained from Wilcoxon-sign rank test.

Nine out of 12 patients demonstrated a clinically significant improvement in 6MWD (>30 m), ranging from 80 m to 170 m from initial to post-training assessment. The distance covered during the 6MWT increased with a group mean of 88 m (95% CI, 52 m to 125 m) (Figure 2a). As a group, patients’ values also increased in relation to age- and gender-specific norm values (Figure 2b,c). The group difference to the LLN increased with 97 m (95% CIs, 59 m to 134 m). Median percentage norm of 6MWD changed from 80% (range 50–103%) to 107% (range 57–125%). Individual data on changes in 6MWT are illustrated in Supplementary Figure S1.

Six out of 10 patients with initially perceived restrictions due to the COVID-19 (PCFS) presented no more restrictions at post-training (PCFS 0). Four patients remained with PCFS scores of 2 and 1. There was a statistically significant improvement in HRQoL from a mean of 65% to 81% (95% CI, 4–25%) on the VAS (0–100%).

## 4. Discussion

From the present cohort of 60 potential candidates, about a third of patients (n = 14) required post-discharge rehabilitation. This is slightly lower than estimated by Murray et al. (2020) [1] and higher than the 10% expected in Greenhalgh et al. (2020) [2]. A final recruitment of 12 out of 60 emphasizes that such outpatient PR is appropriate for a select subgroup of COVID-19 patients point hospitalization. The pre-defined inclusion criteria can be considered as adequate since 75% completed their training (dropout rate of 25%). Nevertheless, it still remains unclear whether the inclusion criteria used in this program were sensitive enough to identify all patients requiring support in an outpatient setting. Predictors for COVID-19 induced deficits in physical performance and HRQoL after hospitalization still have to be determined in research. In addition, exclusion criteria for such outpatient PR remain under discussion. Two patients were excluded from the standard PR program despite evidence indicating that aerobic exercise does not cause adverse consequences in post-infectious chronic fatigue syndrome.

In terms of fatigue symptoms after COVID-19, the optimal approach to treatment is currently under debate. According to the National Institute for Health and Care Excellence (NICE) guidelines from July 2020, managing post-viral fatigue after COVID-19 with graded activity is not recommended [42]. Taking into account the increasing evidence on the high incidence of post-viral fatigue after COVID-19 according to the National Institute for Health Research [43], solid monitoring of fatigue during outpatient rehabilitation is recommended. This should be followed by appropriate medical screening to diagnose post-viral fatigue (i.e., laboratory investigation, pathogen tests) [44].

The interval mode in ACE was tolerated in 83% of patients, and 92% were able to begin their PR at the requested training intensity for ACE. In 83% of patients, progression of exercise intensity was achieved. One adverse event was reported in line with pre-existing musculoskeletal issues. However, none of the patients had training-induced negative events. In the present group, 9/12 increased their 6MWD with a range in post-training difference from 80 m to 170 m after 11 to 24 training sessions. These clinically relevant changes indicate the possible positive effects of the outpatient PR program on recovery.

Furthermore, a retention rate of 100% for follow-up analysis and no missing data are encouraging findings, and point towards a potentially feasible and appropriate outpatient PR program.

### Study Limitations

Some limitations have to be acknowledged for this study. Data were obtained from a small sample, which does not allow for generalizability. Due to the pre-post intervention feasibility study design, no assertions can be made on the causality of the changes observed. Three patients demonstrated changes in 6MWD below 30 m, despite 12 (n = 1) and 24 (n = 2) training sessions. The participant who completed 12 sessions started the program within their normal range of physical performance according to the 6MWT, but with perceived restrictions in this regard. The lack of clinically important change might be due to a ceiling effect. The other patients with a lack of clinically important change to 6MWD were not able to increase intensity progressively because of systemic and musculoskeletal health issues. Furthermore, it should be considered that three patients were included due to perceived deficits in physical performance and limitations in daily life. This could suggest a selection bias emphasizing that no assertions can be made on training effects. Finally, the lack of a control group means that there is no benchmark against which to compare any clinical changes following the intervention.

Whilst acknowledging the above limitations, based on this small number of patients, tolerability of training ranged from 83–100% in terms of training modes and intensity. Adverse events and dropouts were linked with pre-existing musculoskeletal deficits, rather than specific issues with the PR program. This suggests that exercise recommendations for patients with COPD according to Gloeckl and colleagues (2013) [17] can be used in larger trials evaluating outpatient rehabilitation in COVID-19 patients after moderate to critical disease severity. In terms of optimal training duration, there is still a lack of knowledge in the population with COPD [22]. Thus, the duration of rehabilitation was not pre-defined in the present program, because of the novelty of the disease and lack of evidence on training protocols and effects in outpatient rehabilitation after COVID-19. This has to be considered as a limitation in terms of feasibility testing. The present analysis showed that around 70% of patients in the present group required 8–12 weeks to achieve their individual goals in performance and HRQoL. This corresponds with research in PR for patients with COPD [22]. At least 8 weeks are recommended in this population to achieve goals in HRQoL, with a plateau anticipated after 12 weeks. Nevertheless, a comparison of impact in patients with chronic disease and the COVID-19 survivors has to be handled with care. The structural causality for deficits in physical performance in the present group remains unclear and might underlie a large variance. Discrepancies still exist in the literature on the associations between initial disease severity and distance covered at the 6MWT [3,9].

## 5. Conclusions

This feasibility study tentatively suggests that a physiotherapy-guided pulmonary rehabilitation program may be feasible in a select population of patients post hospital discharge following COVID-19. Moreover, such a program has the potential to support patients in achieving patient-tailored goals on performance and HRQoL. The tolerability to moderate interval-based aerobic cycle endurance combined with resistance training was high in this group, who had limitations to their physical performance (real or perceived) after hospitalization. These preliminary findings underpin the relevance of controlled trials investigating the effect and advantage of specific interval-based exercise compared to other approaches.

## Figures and Tables

**Table 1:** Illustration of the training dose and intensity.

Aerobic Cycle Endurance Training (ACE)	Resistance Training (RT)
**Duration** 30 min	**Duration** 30–40 min
**Interval mode** (alternating HI and MI)	
Warm-up 4 min at 15% peakWR HI 4 min 50% peak WR (4×) Borg 4–6 MI 3 min 20–30% peak WR (3×) Cooling-down 3 min at 15% peakWR	10–12 repetitions 50–85% of RM 3 rounds per device Adjustment of weight when more or less than 10–12 repetition achieved
**Continuous mode** 30 min, 20–30% peakWR	

Illustration of the training dose and intensity used in the present study based on the recommendation paper [16]; 50% peakWR of the SRT corresponds 60–80% peak WR recommended by Gloeckl et al. (2013) [16] obtained from cardiopulmonary exercise testing with maximal oxygen uptake. HI = high intensity, MI = Moderate Intensity, RM = repetition maximum.

**Table 2:** Patients’ characteristics.

N = 12	Median (Range); Frequency (Percentage)
Age, years	61 (26–84)
Gender, female (%)	4 (33%)
Severity of pneumonia, n (%)	
Mild	1 (8%)
Moderate	8 (67%)
Severe	2 (16%)
Critical	1 (8%)
Pre-existing Comorbidities	
Cardiovascular disease, n (%)	6 (50%)
Arterial hypertonia, n (%)	3 (25%)
Chronic renal disease, n (%)	5 (n = 5)
Cancerogenous disease, n (%)	3 (25%)
Chronic pulmonary disease, n (%)	2 (16%)
Diabetes mellitus, n (%)	1 (8%)
Adipositas (BMI ≥ 25), n (%)	1 (8%)
Other internal disease, n (%)	2 (16%)
Polyneuropathia, n (%)	1 (8%)
Length of stay at the hospital (days)	11 (3–24)
Duration between COVID-19 diagnosis and PR admission (days)	41.5 (21–73)
Initial 6MWD %Norm, (%)	79.5 (50–100)
Desaturation during 6MWT, yes (%)	4 (33%)
mMRC Dyspnea (0–4)	
0, n (%)	1 (8%)
1, n (%)	4 (33%)
2, n (%)	7 (58%)
3, n (%)	0 (0%)
4, n (%)	0 (0%)
EQ-5D-5L VAS, 0–100% (%)	70 (30–85)
EQ-5D-5L VAS < 80%, n (%)	9 (75%)
Initial PCFS ≥ 2, n (%)	10 (83%)

Values are presented in median and range or median and frequencies except when indicated otherwise. Abbreviations: n = number;BMI = Body Mass Index; PR = pulmonary rehabilitation; 6MWD%Norm = age-gender specific norm value in percentage of the distances covered during 6-min walk test; 6MWT = 6-min walk test; modified medical research council (mMRC); EQ-5D-5L VAS = 5-level Euroqol EQ-5D visual analogue scale; PCFS = post-coronavirus disease 2019 (COVID19)-Functional Status scale; mMRC dyspnea scale (0 dyspnea only with strenuous exercise; 1, shortness of breath when hurrying on the level or walking up a slight hill; 2, walks slower than people of same age on the level because of breathlessness or has to stop to catch breath when walking at their own pace on the level; 3 stops for breath after 91 m walking or after a few minutes; 4 too dyspneic to leave the house or breathless when dressing (Mahler et al., 1988); PCFS (0 = no functional limitations, 1 = negligible functional limitations, 2 = slight functional limitations, 3 = moderate functional limitations, 4 = severe functional limitations).

## Data Availability

The data presented in this study are available on request from the corresponding author. The data are not publicly available due to privacy regulations.

## Supplementary Materials

The following are available online at https://www.mdpi.com/article/10.3390/ijerph18083978/s1, Figure S1: Supplementary Figure S1_individual data6MWD.
