# Supplementary material for: Feasibility of an Outpatient Training Program after COVID-19
Source: Int J Environ Res Public Health. 2021 Apr 9;18(8):3978. doi: 10.3390/ijerph18083978 (PMC8069591; doi:10.3390/ijerph18083978)
Supplement: Supplementary file 1 [file ijerph-18-03978-s001.pdf]

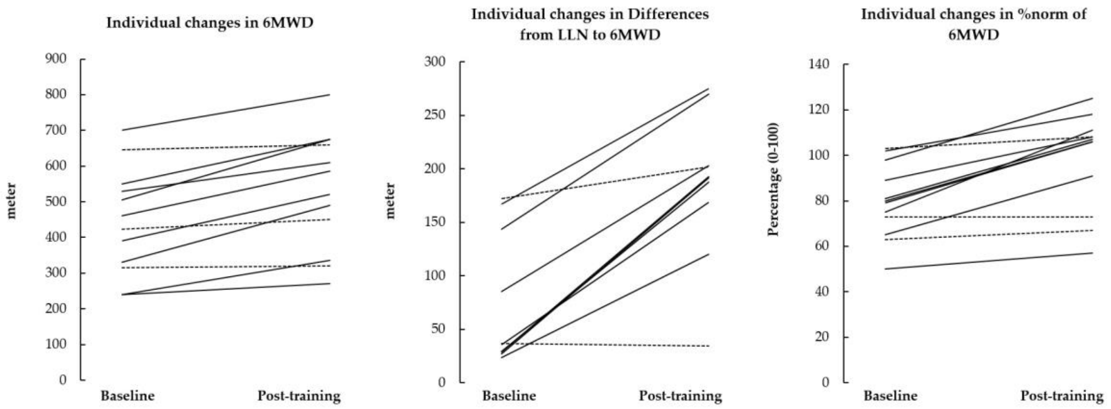

Supplementary figure: paired line graphs with individual changes (n=12) from baseline to post-training assessment in (a) distance covered during the 6-minute walk test (6MWD), (b) difference in Lower Limit of Normal to 6MWD and (c)percentage of age- and gender-related norm values; values are presented in meter or percentage.
